# Determinants of Adverse Birth Outcome in The West Shewa Zone, Oromia, Regional State, Ethiopia: Unmatched Case-control Study

**DOI:** 10.34763/jmotherandchild.20212501.d-21-00003

**Published:** 2021-10-11

**Authors:** Daniel Belema Fekene, Gizachew Abdissa Bulto, Benyam Seifu Woldeyes, Gurmesa Daba Dina, Kassa Mamo Negash

**Affiliations:** 1 Department of Midwifery, College of Medicine and Health Sciences, Ambo University, Ambo, Ethiopia

**Keywords:** Determinant, Adverse birth outcome, West Shewa zone, Ambo, Ethiopia

## Abstract

**Introduction:**

Adverse birth outcome (ABO) can lead to higher rates of poor health and infection for newborns, as well as long-term neurological and health problems. Hence, the aim is to identify determinants of ABOs among mothers who gave birth in hospitals in West Shewa zone, Ethiopia.

**Material and methods:**

A hospital-based, unmatched, case-control study was conducted from March 5 to July 29, 2020, among 591 mothers (171 cases and 420 controls) who had given birth in hospitals found in West Shewa zone. The questionnaire was collected using census and survey processing system (CS-Pro) version 7.1. The data were entered into Epi-data version 3.1 and analyzed by SPSS software version 23. Descriptive statistics, bivariate analysis, and multivariate logistic regression analysis were performed. Finally, P-value <0.05 was used to declare and include variables with statistically significant in predicting the outcome variable.

**Result:**

On multivariate analysis, urban residence (AOR=0.65, 95%, CI=0.43–0.98), lack of family support during child bearing (AOR =5.24, 95% CI=3.16–8.71), pregnancy type (AOR = 4.02, 95% CI: 2.47–6.52), short interpregnancy interval (AOR = 1.43,95% CI= 1.23–4.48), less than four antenatal care (ANC) visits (AOR =1.80,95%CI: 1.17–2.78), and having current obstetric complication (AOR=2.07, 95% CI =1.18–3.61) were significantly associated with adverse birth outcomes.

**Conclusions:**

Residence, lack of family support during childbearing, pregnancy type, short inter-pregnancy interval, having current obstetric complications, and number of ANC visits were identified as determinants of adverse birth outcome. Therefore, improving family support, increasing inter-pregnancy interval through family planning counselling and provision, and having the recommended ANC follow-up were recommended.

## Background

An adverse birth outcome (ABO), which includes preterm births (PTB) and low birth weight (LBW), are major drivers of morbidity and mortality in neonates and infants [1, 2, 3]. ABO is also an important contributor to serious, short- and longterm, physical and mental disabilities, including perinatal and infant death; chronic health problems later in life, such as hypertension, ischemic heart disease, metabolic syndrome, stroke, diabetes, malignancies, osteoarthritis, and dementia; learning difficulties; and hearing and visual impairments [4, 5, 6, 7]. *Preterm* is defined as a baby born alive before 37 weeks of pregnancy are completed [8]. Low birth weight is defined by the World Health Organization (WHO) as a weight of less than 2,500 grams for a live-born infant at birth [8].The majority of severe adverse outcomes during pregnancy and chldbirth result in the death of the mother or her offspring [9,10]. Globally in 2019, 2.4 million children died in the first month of life and about 6,700 neonatal deaths occurred every day; the first 28 days of life were the most vulnerable time for children under age 5 [11]. Regionally, the neonatal mortality rate is highest in Sub-Saharan Africa, followed by Central and Southern Asia. A child born in Sub-Saharan Africa is 10 times more likely to die in the first month of life than a child born in a high-income country and 12 times more likely to die than a child born in Australia or New Zealand [11]. ABOs are influenced by a different of biological, psychosocial, and environmental factors [12, 13, 14]. Different studies indicated that socioeconomic status, maternal education, marital status, pregnancy desire and teenage pregnancy, maternal comorbidities, and genetic vulnerabilities are also linked to poor pregnancy outcomes. Moreover, low pre-pregnancy body mass index (BMI), inadequate weight gain, and poor prenatal care utilization, female foetus, and self-reported cigarette smoking history are related to poor birth outcomes [15, 16, 17]. A high level of anxiety and depressive symptoms during childbirth and pregnancy have been related to a higher risk of adverse birth outcomes [10,14].

In Ethiopia, different studies have shown that the prevalence of ABOs is within the range of 13.9% to 37.6% [18, 19, 20, 21, 22, 23, 24]. Antenatal care (ANC) follow-up, rural residency, pregnancyinduced hypertension, advanced maternal age, current pregnancy complications, anaemia, and twin pregnancy were factors associated with ABOs [18, 19, 20, 21, 22, 23, 24, 25]. Though a few earlier studies were conducted in Ethiopia, those studies were cross-sectional and therefore weak in identifying those factors; those studies were also mainly conducted in single town or district [21,24,26, 27, 28]. Additionally, no other study has been conducted on determinants of ABOs in the study area. Therefore, the current study aimed at identifying determinants of adverse birth outcomes in the West Shewa zone, Oromia, regional state, Ethiopia, by using unmatched case-control study design.

## Material and methods

### Study area and period

The study was conducted in hospitals found in West Shewa, Oromia, region, Ethiopia, from March 5 to July 29, 2020. Ambo town is the capital city of the West Shewa zone, which is located 114 kilometres to the west of Addis Ababa. West Shewa zone has 8 public hospitals, 96 government health centres, 526 health posts, and 77 private clinics. The total population in the zone is 2,058,676, of whom 1,030,175 were females.

### Study design and population

A hospital-based, unmatched, case-control study was conducted. All mothers who gave birth in hospitals in the zone were our source population. Cases were mothers with adverse birth outcome, including preterm birth, low birth weight, or stillbirth; controls were mothers with live births whose infants had birth weights greater than 2,500 grams at birth and were born at term.

### Sample size determination and sampling procedure

The sample size was calculated by Epi info stat calc software. The proportion of mothers having complications during childbirth among controls was used to determine the sample size from the study conducted in Jimma [29]. The assumptions for the sample size calculation were as follows: The proportion of mothers having complications during childbirth among controls 19.6%, odds ratio of 2.9, 95% CI, power level of 80%, and a case-to-control ratio of 1:2. The maximum sample size was 591, of which 171 were cases and 420 were controls. All government hospitals providing 24 hours delivery services in the West Shewa zone were included in the study. The number of cases and controls were proportionally allocated to each hospital based on their last quarter institutional delivery performance report prior to data collection time. Finally, the eligible case was selected consecutively, and three controls were selected consecutively until the required sample size was achieved.

### Data collection tool, quality control, and measurements

A structured interview questionnaire in English was prepared and translated into the local language, Afan Oromo, by the translator; it was then translated back into English by a third person to check for consistency. The questionnaire gathers information on sociodemographic characteristics, past obstetric and gynaecologic experiences, the current obstetric experience, and the characteristics of the newborn at birth; it was adapted from the EDHS (Ethiopian Demographic and Health Survey) and other reviewed literature and modified according to the local context [9,18,20, 21, 22, 23, 24, 25,30]. The data were collected from the mothers, and measurements were taken from the neonates. The questionnaire template was coded using open-source software for Computer Assisted Personal Interviewing and census and survey processing system (CS-Pro) version 7.1 and was deployed to census and survey entry (CS-Entry) android application. Eight nurses were recruited as data collectors and four assistant professors were hired as supervisors. In addition, the data collectors were trained for two days on the techniques of data collection and the purpose of the study for study participants. Pretesting was done on 5% of the total study participants, and necessary adjustments were made to specific word use and sequencing of questions. The weight of the newborns was measured to the nearest 100 grams using a baby measuring weight scale within 15 minutes after delivery.

### Data processing and analysis

The data were collected using CS-Entry for the android version and exported to SPSS version 23 for analysis. Binary logistic regression analysis was done to evaluate the association of ABOs with each independent variable separately. Variables with a p-value <0.2 were entered into the multivariable logistic regression models. Model fitness was tested with Hosmer-Lemeshow goodness of fit test. Furthermore, multicollinearity was checked between the independent variables and (VIF), and tolerance to test multicollinearity and VIF (variance inflation factors) was less than 10 and tolerance >0. Finally, the strength of association was measured by both crude and adjusted odds ratios, with a 95% CI for exposure variables and ABOs. The statistical significance level was declared at p-value < 0.05.

## Result

### Sociodemographic characteristics of mother

A total of 591 mothers (171 cases and 420 controls) were included, with a response rate of 100%. Concerning the educational status of mothers, 56 (32.7%) of the cases are unable to read and write, and 88 (21.0%) of the controls have college or above educations. Of the controls, 118 (28.1%) were farmers 60 (35.1%) of the cases stated their occupations as housewife or mother. The majority of the cases (62.6%) and the controls (58.7%) were Protestant by religion. Ninety (52.6%) of the cases and 164 (28.8%) of the controls were lived in rural areas. (Table 1)

**Table 1:** Sociodemographic characteristics of mothers who gave birth in public hospitals, West Shewa zone, Ethiopia, 2020

Variables	Categories	Case		Control		Statistics (X^2^), p-value
		Frequency	Percentages %	Frequency	Percentages %	
**Age**	<= 23	51	29.8	118	28.1	X^2^=8.45, p=0.04
	24-26	33	19.3	110	26.2	
	27-30	60	35.1	105	25.0	
	31+	27	15.8	87	20.7	
**Religion**	Orthodox	53	31.0	138	32.9	X^2^=1.16, p=0.56
	Muslim	11	6.4	36	8.6	
	Protestant	107	62.6	246	58.7	
**Ethnicity**	Oromo	162	94.7	393	93.6	X^2^=0.29, p=0.59
	Amhara	9	5.3	27	6.4	
**Residence**	Rural	90	52.6	164	39.0	X^2^=9.15, p=0.00
	Urban	81	47.4	256	61.0	
**Mother’s education**	No formal education	56	32.7	140	33.3	X^2^=7.27, p=0.06
	Primary education (1-8)	60	35.1	105	25.0	
	Secondary education (9-12)	27	15.8	87	20.7	
	Collage and above	28	16.4	88	21.0	
**Father’s education**	No formal education	41	24.0	99	23.6	X^2^=8.62, p=0.04
	Primary education (1-8)	56	32.7	93	22.1	
	Secondary education (9-12)	36	21.1	102	24.3	
	Collage and above	38	22.2	126	30.0	
**Occupation of mother**	Government employee	19	11.1	54	12.9	X^2^=2.64, p=0.65
	Private employee Farmer	13 54	7.6 31.6	37 118	8.8 28.1	
	Merchant	25	14.6	48	11.4	
	Housewife	60	35.1	163	38.8	
**Occupation of father**	Government employee	40	23.4	133	31.7	X^2^=5.81, p=0.12
	Private employee	19	11.1	56	13.3	
	Farmer	81	47.4	162	38.6	
	Merchant	31	18.1	69	16.4	
Monthly income	<= 1000	99	57.9	148	35.2	X^2^=7.14, p=0.68
	1,001-3,000	42	24.6	133	31.7	
	3,001+	30	17.5	139	33.1	

### Past obstetric and gynaecologic characteristics of participants

The result of this study shows that 12.3% of cases and 6.0% of the control had a record of pre-existing medical illness, with 35 (20.5) cases experiencing anaemia. Forty-nine (11.7%) of the control and 23 (13.5%) of the cases had histories of abortion in their past pregnancies. Concerning family planning, 117 (68.4%) of cases and 275 (65.5%) of controls cases use family planning for birth spacing (Table 2)

**Table 2:** Past obstetric and gynaecologic characteristics of participants in public hospitals, West Shewa zone, Ethiopia, 2020

Variables	Categories	Case		Control		Statistics (X^2^), p-value
		Frequency	Percentages %	Frequency	Percentages%	
**Abortion history**	Yes	23	13.5	49	11.7	X^2^=0.04, p=0.84
	No	86	50.3	194	46.2	
**Reason for abortion**	Spontaneous	20	11.7	44	10.5	X^2^=2.33, p=0.31
	Medically induced	3	1.8	3	0.7	
	Medical and MVA*	0	-	3	0.7	
**Low birth weight**	Yes	29	17.0	22	5.2	X^2^=18.9, p=0.00
	No	83	48.5	224	53.3	
**Stillbirth**	Yes	8	4.7	27	6.4	X^2^=1.28, p=0.26
	No	104	60.8	219	52.1	
**Preterm**	Yes	31	18.1	23	5.5	X^2^=20.19, p=0.00
	No	81	47.4	223	53.1	
**Ever used family planning method**	Yes	117	68.4	275	65.5	X^2^=0.47, p=0.49
	No	54	31.6	145	34.5	
**Type of family planning method used**	Oral contraceptives	12	7.0	46	11.0	X^2^=5.23, p=0.26
	Implant	37	21.6	74	17.6	
	Injection	60	35.1	136	32.4	
	IUD	6	3.5	18	4.3	
**Medical disorder**	Yes	21	12.3	25	6.0	X^2^=6.19, p=0.01
	No	93	54.4	225	53.6	
**Diabetes mellitus**	No	141	82.5	410	97.6	X^2^=44.27, p=0.00
	Yes	30	17.5	10	2.4	
**Hypertension**	No	130	76.0	334	79.5	X^2^=0.88, p=0.35
	Yes	41	24.0	86	20.5	
**Anaemia**	No	136	79.5	364	86.7	X^2^=0.65, p=0.42
	Yes	35	20.5	74	17.6	

MVA=manual vacuum aspiration IUD=*Intrauterine Device*

### Current obstetric characteristics of participants

Fifty-nine (34.5%) cases had history of one-time pregnancy, and 113 (26.9%) of controls had four or more pregnancies. In terms of planning, 138 (80.7%) cases and 351 (83.6%) of the controls had planned the current pregnancy. A higher proportion of cases and controls didn’t develop complications during the current pregnancy. Fifty-one (29.8%) of the cases and 186 (44.3%) of the controls attended four or more ANC visits. (Table 3)

**Table 3:** Current obstetric characteristics of mothers who gave birth in public hospitals in West Shewa zone, Ethiopia, 2020

Variables	Categories	Case		Control		Statistics (X^2^), p-value
		Frequency	Percentages %	Frequency	Percentages%	
**Gravidity**	<= 1	59	34.5	174	41.4	X^2^=2.95, p=0.23
	2 - 3	65	38.0	133	31.7	
	>_4	47	27.5	113	26.9	
**Antenatal care (ANC)**	Yes	138	80.7	362	86.2	X^2^=2.81, p=0.09
	No	33	19.3	58	13.8	
**Number of ANC visits**	<4	120	70.2	234	55.7	X^2^=2.81, p=0.00
	>_4	51	29.8	186	44.3	
**Current obstetric complication**	Yes	23	13.5	36	8.6	X^2^=3.27, p=0.07
	No	148	86.5	384	91.4	
**Vaginal bleeding**	No	165	96.5	403	96.0	X^2^=0.09, p=0.76
	Yes	6	3.5	17	4.0	
**Obstructed labour**	No	166	97.1	411	97.9	X^2^=0.09, p=0.57
	Yes	5	2.9	9	2.1	
**Anaemia**	No	168	98.2	410	97.6	X^2^=0.22, p=0.64
	Yes	3	1.8	10	2.4	
**Foul smelling discharge**	No	168	98.8	415	98.8	X^2^=0.29, p=0.59
	Yes	3	1.2	5	1.2	
**Birth interval**	< 2 years	107	62.6	198	47.1	X^2^=11.58, p=0.001
	> 2 years	64	37.4	222	52.9	
**Pregnancy planned**	Yes	138	80.7	351	83.6	X^2^=0.71, p=0.43
	No	33	19.3	69	16.4	
**Pregnancy supported by husband**	Yes	162	94.7	402	95.7	X^2^=0.27, p=0.61
	No	9	5.3	18	4.3	
**Family support during pregnancy**	Yes	105	61.4	386	91.9	X^2^=80.42, p=0.00
	No	66	38.6	34	8.1	
**Place of delivery**	Home	11	6.4	27	6.4	X^2^=6.64, p=0.08
	Health Centre	47	27.5	84	20.0	
	Hospital	113	66.1	309	73.6	
**Mode of delivery**	SVD	131	76.6	303	72.1	X^2^=1.56, p=0.46
	Assisted vaginal delivery	7	4.1	16	3.8	
	CS	33	19.3	101	24.0	
**Type of pregnancy**	Single	110	18.6	372	62.9	X^2^=47.48, p=0.00
	Twin	61	10.3	48	8.1	
**Labour onset**	Spontaneous	144	84.2	364	86.7	X^2^=0.61, p=0.43
	Induced	27	15.8	56	13.3	
**Rhesus factor (Rh)**	Positive	158	92.4	391	93.1	X^2^=0.09, p=0.77
	Negative	13	7.6	29	6.9	
**Received tetanus injection**	Yes	98	57.3	253	60.2	X^2^=0.43, p=0.51
	No	73	42.7	167	39.8	

SVD =spontaneous vaginal delivery CS=Cesarean delivery

### Neonatal assessment after birth

The neonatal assessment results indicated that 57.9% of the cases and 65.7% of the controls were male. The first minute APGAR score showed that 25.1% of neonates among the cases and 23.6% among the controls were severely asphyxiated. While 115 (67.3%) of the cases cried at birth, 56 (32.7%) didn’t cry. The percentage of cases receiving skin-to-skin was 82.5%; the percentage of controls receiving such care was 77.9 %. (Table4)

**Table 4:** Neonatal assessments at birth in West Shewa zone, Ethiopia, 2020

Variables	Categories	Case		Control		Statistics (X^2^),p-value
		Frequency	Percentages %	Frequency	Percentages %	
**Sex**	Male	99	57.9	276	65.7	X2=3.23,p=0.07
	Female	72	42.2	144	34.3	
**APGAR score in first minutes**	Normal	55	32.2	160	38.1	X2=1.88,p=0.39
	Moderate asphyxia	73	42.7	161	38.3	
	Severe asphyxia	43	25.1	99	23.6	
**APGAR score in fifth minute**	Normal	97	56.7	253	60.2	X2=1.08,p=0.58
	Moderate asphyxia	34	19.9	69	16.4	
	Severe asphyxia	40	23.4	98	23.3	
**Gestational age**	Preterm	38	6.4	83	14.0	X2=0.45,p=0.50
	Term	133	22.5	337	57.0	
**Birth weight**	Low birth weight	61	35.7	23	3.9	X2=90.87,p=0.00
	Normal birth weight	110	64.3	397	67.2	
**Birth injury**	Yes	2	1.2	11	2.6	X2=1.17,p=0.28
	No	169	98.8	409	97.4	
**Cry immediately after birth**	Yes	115	67.3	306	72.9	X2=1.86,p=0.17
	No	56	32.7	114	27.1	
**Skin-to-skin contact**	Yes	131	22.2	327	77.9	X2=0.10,p=0.74
	No	30	6.8	93	22.1	
**Breastfeeding within one hour**	Yes	62	36.3	186	44.3	X2=3.25,p=0.07
	No	109	63.7	234	55.7	
**Provided first initial newborn care**	No	70	40.9	265	63.1	X2=24.30,p=0.00
	Yes	101	59.1	155	36.9	

### Determinants of adverse birth outcome

Bivariate logistic regression analysis was performed using odds ratios (OR) and 95% CI. The predictor variables with p-value less than 0.2 in the bivariate logistic regression analysis were entered into the multivariable logistic regression analysis model to control the influence of potential confounding variables. The correlation between the independent variables was checked.

After controlling for confounders using multivariable analysis residence, lack of family support during childbearing, pregnancy type, short inter-pregnancy interval, current obstetric complications, and number of ANC visits were identified as determinants of ABOs. (Table 5)

**Table 5:** Bivariate and multivariate logistic regression analysis of determinants of adverse birth outcome in West Shewa zone, Ethiopia, 2020

Variables	Adverse birth outcome		COR95%CI		P-value
	Yes	No		AOR95%CI	
**Residence**					
**Urban**	81(13.7%)	256(43.3%)	0.57 (0.403-0 .83)	0.65(0.43-0.98)*	0.040
**Rural**	90(15.2%)	164(27.7%)	1	1	
**Family support during childbearing**					
**No**	66(11.2%)	34(5.8%)	7.14 (4.47-11.38)	5.24(3.16-8.71)*	0.000
**Yes**	105 (17.8%)	386(65.3%)	1	1	
**Pregnancy type**					
**Twins**	61(10.3%)	48(8.1%)	4.29(2.78-6.63)	4.02 (2.47-6.52)*	0.000
**Single**	110 (18.6%)	372 (62.9%)	1	1	
**Birth interval**					
**<2 years**	107(18.1%)	198(33.5%)	1.87 (1.30-2.69)	1.43(1.23-4.48)*	0.0001
**>2 years**	64 (10.8%)	222(37.6%)	1	1	
**Number of antenatal care visits**					
**<4**	120(20.3%)	234(39.6%)	1.87(1.28-2.74)	1.80(1.17-2.78)*	0.008
**>=4**	51(8.6%)	186(31.5%)	1	1	
**Current obstetrics complication**					
**Yes**	43(7.3%)	39(6.6%)	3.28(2.04-5.3)	2.072(1.18-3.61)*	0.001
**No**	128(21.7%)	381(64.5%)	1	1	

Keys: 1=Reference category *Statistically significant at p<0.05 in multivariate

Mothers who live in urban areas are 1.5 times less likely to develop ABOsas compared with women living in rural areas (AOR=0.65, 95%, CI=0.43-0.98). The odds of having adverse birth outcomes increases twofold for mothers whose birth intervals are less than two years, as compared to their respective referent group (AOR_=_1.43,95% CI=1.23-4.48). Furthermore, mothers who have no family support during childbearing had a five times greater chance to develop ABOs as compared with mothers who had family support (AOR =5.24, 95% CI=3.16-8.71). The number of antenatal care visits was found to be associated with the incidence of ABOs, with mothers who had fewer than four antenatal care visits being twice as likely to experience adverse birth outcomes as compared to their counterparts with four or more such visits (AOR = 1.80, 95% CI: 1.17-2.78). Mothers who gave birth to twins had a four times greater chance of an ABO than mothers who gave birth to a singleton (AOR = 4.02, 95% CI: 2.47-6.52). Mothers having current obstetric complications were twice as likely to experience adverse birth outcomes as compared to mothers with no current obstetric complications (AOR=2.07, 95% CI: 1.18-3.61).

## Discussion

This study tried to identify determinants of ABOs among mothers who delivered in hospitals in West Shewa zone. Women’s place of residence was found to be significantly associated with ABOs. Those women residing in urban areas were 1.5% less likely to develop ABOs than those in rural areas, a result similar to that found in studies reported elsewhere in Ethiopia (in Gamo Gofa zone, Hosana town, and northern Wollo) and in China [22, 23, 24, 25]. This could be due to the relative lack of access in rural areas to quality pregnancy-related care, including medical services, health information, and nutritional awareness.

The number of antenatal care (ANC) visits is significantly associated with ABOs: mothers who had fewer than four ANC visits were twice as likely to have ABOs as those who had four or more such visits. This finding is supported by studies conducted in Cameroon, India, Malawi, Addis Ababa, and in Ethiopia, in Tigrai region, Amhara region,and North Shewa zone [31, 32, 33, 34, 35, 36, 37]. This might be because mothers who have four or more ANC visits gain access to different or additional health promotion and preventive interventions that enhance the health of both the mother and foetus.

Having a history of current obstetric complications was also found to be significantly associated with ABOs. The chance of developing an abnormal birth outcome among mothers with current histories of child-related abnormal birth outcome was twice as high as the chances of mothers without such complications. A study conducted in Gambia and Nigeria showed that mothers with a current history of child-related abnormal birth outcomes are at greater risk of giving birth to a baby with abnormal outcomes [38,39]. Similar findings were previously reported in Ethiopia [21,23,24]. The link may be explained by the impact on the well-being of the foetus in the uterus of complications affecting the mother during pregnancy [40].

Mothers who have no support during childbearing had a five times greater chance of developing adverse birth outcomes as compared to mothers who have partner support. This study was in line with a study done in the United States that found that women with a supportive partner were 63% less likely to have low birth weight infants and nearly two times less likely to have a pregnancy loss, as compared to those with no partner support [30]. Those who have paternal support may experience less stress and thus be more likely to enter prenatal care; they may also be more likely to report a desired pregnancy, which may also reduce their risk of poor birth outcomes.

Short inter-pregnancy interval is also found to be a determinant of ABOs. The odds of having an ABO were 1.43 times greater among mothers with short birth intervals, as compared to mothers having optimal birth spacing. This result is in line with studies in Tanzania; California, Ohio, and elsewhere across the United States; and Bangladesh [41, 42, 43, 44], which showed short inter-pregnancy intervals were a risk factor for low birth weight and/or preterm birth. For example, a study conducted in Tanzania found that women who conceived at either shorter (less <24 months) or longer (37 to 59 months or more) inter-pregnancy intervals had a greater risk of preterm birth [41], and studies conducted in California, Ohio, and elsewhere across the United States showed that intervals shorter than 6 months might be associated with increased risk of adverse outcomes in the subsequent pregnancy [42,43]. Study results from Bangladesh showed that a very short birth interval less than 21 months (birth-to-pregnancy of less than 12 months when pregnancy is carried to term) is associated with an increased risk of adverse pregnancy outcomes, but intervals of 24 to 32 months (birth-to-pregnancy interval of 12 to 23 months when pregnancy is carried to term) and 33 to 44 months (birth-to-pregnancy interval of 24 to 35 months) do not appear to be [44]. This could be because short inter-pregnancy interval results in maternal nutrition reduction, which compromises the mother’s ability to support foetal growth and development, which in turn increases the risks of preterm birth, growth restriction, and maternal morbidity and mortality in the subsequent pregnancy [40,45].

In this study, mothers having current obstetric complications were three times more likely to develop adverse birth outcomes as compared to mothers with no history of current complications. This result is supported by studies conducted in Gondar, Ethiopia; Hosanna, Ethiopia; and a university and hospital in Nashik, India. [19,23,32].

## Conclusions

The result of this study revealed that residence, lack of family support during childbearing, pregnancy type, short inter-pregnancy interval, current obstetric complications, and a number of ANC visits were determinants of adverse birth outcome. Therefore, improving family support, inter-pregnancy intervals through family planning counselling and provision, having the recommended ANC visits, were recommended.

## Abbreviations

ABO: Adverse birth outcome
ANC: Antenatal care
AOR: Adjusted odds ratio
CI: Confidence interval
COR: Crude odds ratio
GA: Gestational age
LBW: Low birth weight
MM: Maternal mortality
PTB: Preterm birth
SD: Standard deviation
WHO: World Health Organization
